# Dataset: a social accounting matrix for Germany

**DOI:** 10.1186/s13104-024-06925-2

**Published:** 2024-09-12

**Authors:** Kevin Connolly, Andrew Ross, Stefan Vögele

**Affiliations:** 1 Department of Economics, Fraser of Allander Institute, University of Strathclyde, Glasgow, Scotland; 2 Institute of Climate and Energy Systems, Jülich Systems Analysis, Forschungszentrum Jülich GmbH, 52425 Jülich, Germany

**Keywords:** National Accounts, Social Accounting Matrix, Computable General Equilibrium, Input-Output

## Abstract

**Objectives:**

The Social Accounting Matrix (SAM) is an extension of Input-Output tables that records macro and meso-economic accounts of a socio-economic system. Its main objective is to provide a comprehensive understanding of the interrelationships among different economic sectors and agents. The SAM can be used for various purposes, including economic analysis, policy evaluation, and economic modelling. It allows policymakers to make more informed decisions, understand potential consequences of different policy options and serve as the foundation for constructing Computable General Equilibrium (CGE) models.

**Data description:**

The SAM for Germany is a comprehensive source of data that reveals the incomes and expenditures of 163 different production sectors, along with data on factors of production, households, corporations, government, and external accounts with the rest of the world. Additionally, it provides information on gross fixed capital formation, changes in inventories, and natural capital accounts. This SAM was compiled by extending the EXIOBASE Input-Output (IO) accounts with data from the Federal Statistical Office of Germany. Balancing items were also used to ensure that the Total Income and Total Expenditure of the main transactors are in balance.

## Objective

The SAM is an extension of IO tables that records macro and meso-economic accounts of a socio-economic system [1]. Unlike IO tables, the SAM records flows from producing sectors to factors of production, institutional accounts, and then back to the demand for goods, and so it can improve upon the Type II IO multiplier [2]. Additionally, the SAM contains complete information on institutional accounts such as households, government, and corporations, while IO tables only trace income and expenditure flows associated with the production of commodities [3].

The SAM offers added value by serving various purposes, including economic analysis, policy evaluation, and economic modelling [1, 3, 4]. Specifically, it provides a comprehensive overview of the structure of the German economy and highlights the relationships among different sectors and economic agents, allowing for a more complete understanding of the economy. The SAM can also be used to assess the potential impacts of various economic policies related to income distribution, employment, and economic growth, assisting policymakers in making more informed decisions and understanding the potential consequences of different policy options. Moreover, the SAM serves as foundational data for constructing computable general equilibrium (CGE) models, which assist in understanding how the economy might respond to different policy changes or external shocks.

In other words, the SAM provides a comprehensive framework that allows policymakers and researchers to better identify interrelationships among different economic sectors and agents. The significance of this kind of data is demonstrated in recent studies, where it has been employed to evaluate the socio-economic consequences of climate change-induced water scarcity [5], and to identify the system-wide impacts of labour-augmenting technological change [6], for example.

## Data description

Table 1 gives an overview of data files. The file DE_SAM_2020_IXI.xlsx gives the SAM for Germany, whilst the file DE_SAM_OVERVIEW.xlsx outlines the structure and dimensions of the SAM. The SAM includes information on the incomes and expenditures that take place within the German economy. It includes data on 163 production sectors (activities), factors of production (LAB and CAP), indirect business taxes (IBT), households (HH), corporations (FIRMS), and government (GOV). Additionally, it provides details on gross fixed capital formation (KFOR), changes in inventories (STOCK), as well as the external accounts with the rest of the world (ROW). The capital accounts are divided into subcategories based on water and land resources to create the natural capital accounts. The data are presented in a 173 × 173 square matrix format and are provided in a single worksheet. All figures are reported in billions of euros for the year 2020, although the data were compiled for other years, but these are not included in this listing.

The compilation of the SAM follows the approach outlined in Emonts-Holley, et al. [7], where it was first applied for Scotland and then later for the UK. This approach allows for complete incorporation of national/regional IO accounts. That is, the Activities accounts include all the relevant parts of the EXIOBASE Input-Output [8] accounts, without any modifications made during the balancing or compilation process. In other words, these data are presented exactly as they are in the original source.

The SAM is formed by extending the EXIOBASE IO [8] accounts with data from DESTATIS [9]. Remaining entries are balancing items (or the sum of entries). As outlined in Emonts-Holley, et al. [7], balancing items are used to ensure that the Total Income and Total Expenditure of the main transactors are in balance. For example, the Household and Government accounts have control totals (from DESTATIS), which require manual balancing to ensure that total income is equal to total expenditure. Generally, balancing items are imposed on cells with the least robust data availability or quality. Via this data entry and balancing, a square SAM is produced where in each account the incomes are equal to the expenditures.

**Table 1:** Overview of data files/data sets

Label	Name of data file/data set	File types (file extension)	Data repository and identifier (DOI or accession number)
Data file 1	DE_SAM_2020_IXI Social Accounting Matrix for Germany	MS Excel file (.xlsx)	Zenodo (10.5281/zenodo.7924140) [10]
Data file 2	DE_SAM_Overview Dimensions of theSocial Accounting Matrix for Germany	MS Excel file (.xlsx)	Zenodo (10.5281/zenodo.7924140) [10]

The structure of the SAM presented in Table 2outlines its dimensions and data sources, as denoted by superscripts. Specifically, the superscripts B, D, and E signify the sources and data processing methods used in the SAM. The SAM incorporates data from multiple secondary sources, such as DESTATIS [9] (superscript D), EXIOBASE [8] (superscript E), and balancing items (superscript B). This tabulated structure aids researchers in gaining clearer insights into the foundational data and its compilation process.

**Table 2:** Structure of the social accounting matrix for Germany

	ACTIVITIES	LAB	CAP	IBT	HH	FIRMS	GOV	KFOR	STOCK	ROW	TOTAL
**ACTIVITIES**	163 × 163^E^	-	-	-	163 × 1^E^	-	163 × 1^E^	163 × 1^E^	163 × 1^E^	163 × 1^E^	163 × 1^E^
**LAB**	1 × 163^E^	-	-	-	-	-	-	-	-	-	1 × 1^E^
**CAP**	1 × 163^E^	-	-	-	-	-	-	-	-	-	1 × 1^E^
**IBT**	1 × 163^E^	-	-	-	1 × 1^D^	-	-	1 × 1^B^	-	1 × 1^D^	1 × 1^S^
**HH**	-	1 × 1^E^	1 × 1^D^	-	-	1 × 1^D^	1 × 1^D^	-	-	1 × 1^B^	1 × 1^D^
**FIRMS**	-	-	1 × 1^B^	-	1 × 1^B^	-	1 × 1^D^	-	-	1 × 1^B^	1 × 1^S^
**GOV**	-	-	1 × 1^D^	1 × 1^E^	1 × 1^D^	1 × 1^D^	-	-	-	1 × 1^B^	1 × 1^S^
**KFOR**	-	-	-	-	1 × 1^D^	1 × 1^D^	1 × 1^D^	-	-	1 × 1^B^	1 × 1^S^
**STOCK**	-	-	-	-	-	-	-	1 × 1^E^	-	-	1 × 1^E^
**ROW**	1 × 163^E^				1 × 1^E^	1 × 1^B^	1 × 1^E^	1 × 1^D^	1 × 1E		1 × 1^S^
**TOTAL**	1 × 163^E^	1 × 1^E^	1 × 1^S^	1 × 1^E^	1 × 1^D^	1 × 1^S^	1 × 1^D^	1 × 1^S^	1 × 1E	1 × 1^S^	

Note: (D): exiobase [8] (E), DESTATIS [9] (D), (B) balancing items (B), sum (S)

## Limitations

One of the main advantages of the method proposed by Emonts-Holley, et al. [7], which was employed in the creation of the SAM for Germany, is that is that it keeps the underlying IO table unchanged. This allows for the inclusion of official and published data without modifications. In cases where data are unavailable, balancing items are used to ensure that total income aligns with total expenditure. Nevertheless, a disadvantage of this approach is that the balancing item entries may lack precision. This issue can be mitigated by integrating more accurate data whenever they become available. Therefore, data are updated whenever possible, while ensuring that the fundamental structure of the SAM, as outlined in Table 2, remains intact.

## Data Availability

The data described in this Data note can be freely and openly accessed on Zenodo under 10.5281/zenodo.7804529. Please see Table 1 and [10] for details and links to the data.

## Abbreviations

CGE: Computable General Equilibrium
EXIOBASE: Multi-Regional Environmentally Extended Input-Output Table
IO: Input-Output
SAM: Social Accounting Matrix
